# Vitellogenin and vitellogenin receptor gene expression is associated with male and female parenting in a subsocial insect

**DOI:** 10.1098/rspb.2015.0787

**Published:** 2015-06-22

**Authors:** Eileen M. Roy-Zokan, Christopher B. Cunningham, Lauren E. Hebb, Elizabeth C. McKinney, Allen J. Moore

**Affiliations:** Department of Genetics, University of Georgia, Athens, GA 30602, USA

**Keywords:** burying beetle, ovarian ground plan hypothesis, parental care, reproductive ground plan hypothesis, sociality

## Abstract

Complex social behaviour in Hymenoptera has been hypothesized to evolve by co-opting reproductive pathways (the ovarian ground plan hypothesis, OGPH) and gene networks (the reproductive ground plan hypothesis, RGPH). In support of these hypotheses, in eusocial Hymenoptera where there is reproductive division of labour, the yolk precursor protein vitellogenin (Vg) influences the expression of worker social behaviour. We suggest that co-opting genes involved in reproduction may occur more generally than just in the evolution of eusociality; i.e. underlie earlier stages of social evolution such as the evolution of parental care, given that reproduction and parental care rarely overlap. We therefore examined *vitellogenin* (*vg*) gene expression associated with parental care in the subsocial beetle *Nicrophorus vespilloides*. We found a significant reduction in the expression of *vg* and its receptor, *vgr*, in head tissue during active parental care, and confirmed that the receptor is expressed in the brains of both sexes. Ours is the first study to show that *vgr* is expressed in the brain of a non-eusocial insect. Given the association between behaviour and gene expression in both sexes, and the presence of vitellogenin receptors in the brain, we suggest that Vg was co-opted early in the evolution of sociality to have a regulatory function. This extends the association of Vg in parenting to subsocial species and outside of the Hymenoptera, and supports the hypothesis that the OGPH is general and that heterochrony in gene expression is important in the evolution of social behaviour and precedes subsequent evolutionary specialization of social roles.

## 1. Introduction

We have little information on ‘how’ advanced social behaviour evolves—the genetic, physiological and developmental basis—compared with ‘why’—the nature of selection and fitness consequences of social behaviour [1]. Many of the studies examining the direct effects of specific genes associated with sociality have been conducted on the eusocial insects, especially honeybees (*Apis mellifera*). The defining aspects of eusocial insects are brood care, overlapping generations where offspring assist the parent in caring for young and reproductive division of labour [2]. Although these are highly derived traits, and the honeybee serves as an excellent model to examine the evolution of such traits, the parsimony of evolution suggests that common mechanisms and genetic bases should influence some or all of the related traits in organisms with less highly developed social systems, independently of taxonomic relationships. Here, we examine the role of vitellogenin (Vg), a molecule closely associated with division of labour and behavioural specialization in honeybees [3–6], in an evolutionarily independent subsocial beetle to test the hypothesis that Vg is associated with changes in social behaviour outside of eusociality. We chose to study a subsocial species because in subsocial species, parental care exists but there is no caste specialization as is seen in eusocial species. Subsociality is a behavioural stage that is considered a necessary precursor for the evolution of eusociality [1,2].

The current hypotheses for the mechanisms behind the unique behavioural traits associated with eusociality are the ovarian ground plan hypothesis (OGPH), linking the decoupling of ovarian physiological cycles and behaviour [7], and the reproductive ground plan hypothesis (RGPH) [4] derived from the OGPH that more specifically posits that regulatory gene networks involved in reproduction have been co-opted to facilitate the behavioural differences seen in worker castes. The RGPH led to examinations of the links between the yolk precursor protein Vg levels and behavioural transition from nursing to foraging workers in eusocial insects, where Vg appears to regulate diverse social behaviours and several aspects of life history. While Vg's main function is to provide lipids and carbohydrates to growing embryos within oocytes, it also transports other nutrients, such as metals, to the ovaries [4,8,9]. It is Vg's role as a zinc carrier that protects honeybee workers and queens from oxidative stress [4,10] and contributes to queen longevity [11]. Importantly, *vg* expression also influences behaviour in multiple insects. In honeybees, *vg* gene expression is associated with the division of labour [3–6,12]. This research has shown that worker bees exhibit caste-specific expression patterns of *vg* where nursing individuals (parental care) express *vg* at a much higher levels and have more developed ovaries than foraging workers (food-gathering). Knockdown of *vg* using RNAi shifts workers from nursing to foraging, suggesting a direct link between *vg* expression and division of labour [5]. Vitellogenin also exhibits caste-specific expression pattern and subfunctionalization of specific paralogues within ants [13,14] and has been shown to influence polyphenism (workers or queens) acting as a maternal effect in eggs [15]. In bumblebees, a primitively eusocial species, *vg* expression is more closely associated with social status and aggression than with reproductive state [16]. Although it is clear that this multifunctional protein has been a component in the evolution of eusociality and possibly social interactions, exactly how this gene was co-opted for a role in a division of labour or whether its role in behavioural transition is unique to Hymenoptera is unclear. Is a role for Vg in social interactions more general? How far along in the evolution of sociality did a role for Vg arise?

Here, we tested the specific hypothesis that *vg* expression is associated with the evolution of a behavioural precursor to eusociality, parental care. We test for a role of *vg* in parental care in the burying beetle *Nicrophorus vespilloides*, a subsocial beetle that does not have division of labour but does have extensive parental care involving direct interactions with begging offspring and parental regurgitation of pre-digested food to offspring [17,18]. In division of labour, parental care is the flip side of the food-gathering tasks of workers. Instead of collecting food, parental care involves the distribution of food through regurgitation to siblings or offspring. Our hypothesis is that *vg* is associated with the expression of feeding and parent–offspring interactions, and while it may be further co-opted during the evolution of a division of labour, it is not specific to the evolution of eusociality. Our hypothesis regarding *vg* expression is based on two arguments. First, we expect genes involved in reproduction to be co-opted to influence social interactions such as parental care because parental care and reproduction rarely co-occur. Thus, inferring from the OGPH, co-opting genes associated with reproduction to influence social interactions between parents and offspring is possible and may be general to the evolution of sociality. Second, we specifically targeted Vg because of its role in social insects and its relationship to the RGPH.

Examining this in a burying beetle provides several independent tests of the role of Vg in behaviour. Working with a subsocial insect outside the Hymenoptera expands the taxonomic range, where *vg* expression is examined under a social context. In addition, while many burying beetles may be biparental, parental care in *N. vespilloides* is often uniparental [17,18], and care can be performed by either sex (e.g. in a study in nature of 258 families, 39% were uniparental female, 3% uniparental male [19]; in our study of 269 families, 51% were uniparental female, 5% uniparental male; A.J.M. 2015, unpublished data). This provides us with an opportunity to examine gene expression in both males and females performing care and allows a clean decoupling of the yolk precursor function of Vg from its behavioural influences. Finally, parental care in *N. vespilloides* is not confounded by permanent developmental changes as it is in eusocial insects that transition to new caste with age, and so we can examine both change in expression and the transition back. Work on a parthenogenetically reproducing ant, *Cerapachys biroi* [20], also decoupled behaviour and permanent transitions as this ant cycles between care and reproduction, but a strength of our study is that we investigate *vg* expression and parental care in both males and females. Any differences seen in males are inconsistent with Vg being associated with reproduction, as males do not make eggs. Here, we identify two copies of *vg* genes within *N. vespilloides* and show expression differences in *vg* and its receptor, *vgr*, associated with transition to parental care behaviour in both males and females. Expression levels decrease during care and begin to rise again after the cessation of care. We also find that *vgr* is expressed in the brains of both male and female beetles. These results provide support for the hypothesis that Vg was co-opted into a behavioural role independent of eusociality. This further suggests that OGPH and RGPH are more general and associated with the evolution of sociality and its earlier forms, such as subsocial behaviour.

## 2. Material and methods

### (a) Gene identification

We identified *N. vespilloides vg* and *vgr* genes by running a tBLASTn search on our unpublished genome and transcriptome databases. *Nicrophorus orbicollis* possesses two *vg* genes that have been previously described using this specific motif [21]. Both *N. orbicollis* Vg GL/ICG motif sequences (AAW19633 and AY728385) were used as input for the search for *vg* genes. The GL/ICG motif is a conserved approximately 500 bp region located at the C-terminal end of almost all Vg proteins [22]. Search results returned two contigs that preferentially matched with either Vg1 or Vg2 GL/ICG motif sequences from *N. orbicollis* and primers were designed for the two putative *N. vespilloides vg* genes at the most 5′ and 3′ regions of the sequences. The two putative *vg* nucleotide sequences shared high sequence homology and primers were designed to ensure specificity for the two hypothetical paralogues. Primer sequences are available upon request. We used coding sequences from *Tribolium castaneum* and *Periplaneta americana* to search for VgR within our databases (VgR: XP968903 and BAC02725, respectively). Search results returned nearly full *vgr* sequence and primers were designed to target amplification in overlapping fragments. PCR conditions were as follows: 94°C for 5 min, 40 cycles of 94°C for 30 s, 52°C for 30 s, 72°C for 1 min, and a final extension of 72°C for 5 min. PCR reactions were performed using Phusion High-Fidelity DNA Polymerase (Thermo Scientific). Primer sequences are available upon request. PCR products were sent off for Sanger sequencing to the Georgia Genomics Facility at the University of Georgia. We validated sequencing results using BLASTp in NCBI to confirm sequence similarity to orthologous Vg and VgR sequences from other insects.

### (b) Phylogenetic and evolutionary analyses

Phylogenetic analyses were performed on putative *N. vespilloides vg* and *vgr* sequences for further confirmation of gene identity. Orthologous insect Vg (GL/ICG motif only) and VgR protein sequences were obtained through NCBI using BLASTp. Amino acid sequences were aligned using Clustal W [23] within MEGA v. 5.1 [24] under the BLOSUM protein weight matrix for alignment. Two separate phylogenetic analyses were performed to test for congruence: maximum likelihood and a Bayesian phylogenetic analysis. Model testing and Bayesian inference phylogenetic analysis were conducted in MrBayes v. 3.2 [25]. Using a mixed model approach, the Wag model of protein evolution was found to best fit the data for the three datasets and this model was used for both the Bayesian and maximum-likelihood analyses. The Bayesian phylogenetic analysis was conducted for 5 000 000 generations with a sample frequency of every 500 generations, producing a total of 10 000 trees. Trees were summarized after a burnin of 2500 (25%) trees. Maximum-likelihood analysis was conducted in MEGA 5.1 with a bootstrap parameter of 500.

### (c) Experimental design

All *N. vespilloides* used in this study were maintained under standard burying beetle conditions, with adults kept in separate containers from the wandering larval stage [26,27]. The individuals used in this experiment had been maintained in the laboratory for three to four generations (less than a year). New beetles, collected from the wild in Cornwall, UK, are added to the colony every year. Expression analyses were conducted in age-matched adult beetles (21 days post-adult emergence) experiencing different social conditions. Adult head samples were collected for five different social conditions that represent key biologically relevant events during the transition from pre-care to post-care: virgin, mated without a mouse carcass, mated with a mouse carcass, caring for larvae and post-caring dispersed. These five conditions reflect behavioural and physiological differences: virgin, which reflects a ‘control’ or baseline state. Mated off a mouse provides social interactions but does not stimulate reproduction. Mated on a mouse provides social interactions and stimulates ovarian development in females [28,29] and indirect parental care (carcass maintenance and processing to prevent decay [28–30]). Caring for larvae reflects the condition when males and females are no longer reproducing or mating but instead directly interact with the larvae and feed them regurgitated food, thus providing both direct and indirect care [30]. Post-care reflects a return to a mated state but not yet reproducing. Post-caring, there are no further social interactions, and no further reproduction will occur until another carcass is encountered.

Virgin beetles consisted of males and females that had been isolated since larval dispersal in individual containers. This is a non-social state, as there were no interactions with any other beetle since dispersal as larvae. For the two reproductive social conditions (mated on and off a mouse carcass), males and females were paired for 48 h in a mating box (17.2 × 12.7 × 6.4 cm; Pioneer Plastics, Dixon, KY, USA) half filled with dirt that either contained or did not contain a mouse carcass, respectively. Mated without a mouse condition examines the changes that occur when two individuals must tolerate and interact with each other for the sole purpose of mating. However, females do not fully develop their ovaries, nor lay any eggs, until they are upon a resource [28,29]. With the addition of a mouse carcass, we are measuring the state at which females are preparing to lay eggs and also the state at which the male and female prepare the resource for their larvae; i.e. indirect parental care. Finally, for both the caring and post-caring (non-reproductive) conditions, males and females were placed together in a mating box with a mouse carcass present and allowed to mate for 48 h. After 48 h, one of the sexes was removed after mating to ensure that the individual remaining reflected the conditions leading to uniparental care. Females and males were collected for the caring condition only if they were observed to be directly interacting with the larvae. Post-caring beetles were removed from the carcass 24 h prior to larvae dispersal. They were kept in individual containers for 24 h to ensure that they were past the caring state before being sacrificed.

Two different tissues types were collected for expression analyses: whole heads and, for confirmatory analyses, carefully dissected individual brain tissue. Whole heads were snap frozen immediately in liquid nitrogen and stored at −80°C until time of RNA extraction. Ten heads per sex were collected per social condition. The advantage of this approach is that there is little delay between the preservation of tissue and the behavioural state measured. Single brain dissections were performed with heads submerged in ice-cold 1× PBS (National Diagnostics, Atlanta, GA, USA) and were stored at −20°C in 50 μl of RNAlater (Ambion, Grand Island, NY, USA). Dissections were completed within 5 min of beetle removal from social condition. Five brains per sex were dissected for each social condition.

### (d) Quantitative real-time PCR

Total RNA was extracted from adult head samples using a Qiagen RNAeasy Lipid kit (Qiagen, Venlo, The Netherlands) per manufacturer's instructions with frozen heads initially homogenized in 500 μl of Qiazol in a mortar chilled with liquid nitrogen. Total RNA was extracted from adult single brain samples using a Qiagen RNeasy Micro Kit (Qiagen) with a Qiazol extraction method added to the beginning of the protocol. Brain samples were transferred from RNAlater to a 1.5 ml microcentrifuge tube containing 350 μl of Qiazol and homogenized with a hand-held motorized pestle. An additional 350 μl of Qiazol were added and samples were incubated for 5 min at room temperature. One hundred and forty microlitres of chloroform were added to each sample and incubated for an additional 2 min. Samples were then centrifuged at 4°C at 12 000*g* for 15 min and the upper aqueous solution was transferred to a new microcentrifuge tube. One volume of 70% ethanol was added to each sample and then transferred into RNeasy Mini spin columns. The remaining procedure was as described in the manufacturer's instructions for the Qiagen RNAeasy Lipid kit with the addition of an on-column DNase I digestion (Qiagen). RNA was quantified in 1 : 10 dilutions using a Qubit 2.0 florometer (Invitrogen Corporation, Carlsbad, CA, USA) and cDNA was synthesized from 500 ng of RNA using Quanta Bioscience qScript reverse transcriptase master mix following manufacturer's instructions. RNA samples were stored at −80°C and cDNA samples were stored at −20°C until use.

*Vitellogenin* and *vgr* mRNA levels were quantified by quantitative real-time PCR (qRT-PCR) on a Roche LightCycler 480 platform using Roche LightCycler 480 SYBR I Green Master Mix and a 60°C annealing temperature. Each experiment was run on a single 364-well plate. cDNA was diluted 1 : 10 and 2 μl were used as input into a 10 μl reaction containing 5 μl of SYBR mix and 3 μl of a primer stock containing both sense and antisense primers at 1.33 μmol l^−1^. qRT-PCR primers were designed for the two *vg* paralogues and *vgr* using PrimerQuest at Integrated DNA Technologies. Primer sequences were as follows: Vg1rtSense (5′-CATTGATGCTGGACGTGTTTCT-3′); Vg1rtAnti (5′-TCACGTTCGACATAGCGAGTTT-3′); Vg2rtSense (5′-CGAATGTTTCTACAAGGATAA-3′); Vg2Anti (5′-ATCAGAGTTACGCTGTCCAA-3′); VgRrtSense (5′-AGTGTACGGAGGAAGTCGGG-3′); VgRrtAnti (5′-GGCGTGTCTTCAGAGTGCAA-3′). We ran three technical replicates for each biological sample. A dissociation step was added at the end of each run to verify that only a single amplicon was produced. *vg1* and *vg2* amplicons were sequenced to confirm that the appropriate gene was targeted. TATA-binding protein and alpha-tubulin were used as an endogenous control for all experiments. Primer efficiency calculations, genomic contamination testing and endogenous control gene selection were performed as described by Cunningham *et al*. [27].

### (e) Data analysis

We used the ΔΔ*C*_T_ method to assess gene expression changes associated with behavioural states. Data were graphically displayed as relative expression using ΔΔ*C*_T_ method to facilitate comparisons as relative expression is presented by convention, but statistical analyses performed on log_2_ data, which were all normally distributed, whereas relative expression was not. We report all pairwise post hoc comparisons, using Fisher's LSD pairwise comparisons, alongside the overall ANOVA. Fisher's LSD preserves the experimentwise error rate and does not protect against familywise error if there are more than three treatments, so we also made specific pairwise contrasts using Dunnett's method, comparing expression of each state to virgin (baseline) levels of expression. Both analyses gave similar results so we present just the Fisher's LSD results. All analyses were made using JMP Pro (v. 11.0.0). All data used in the analyses are available in Dryad so that our analyses can be checked and verified if desired.

## 3. Results

### (a) Gene identification within the *Nicrophorus vespilloides* genome

**Figure 1.**
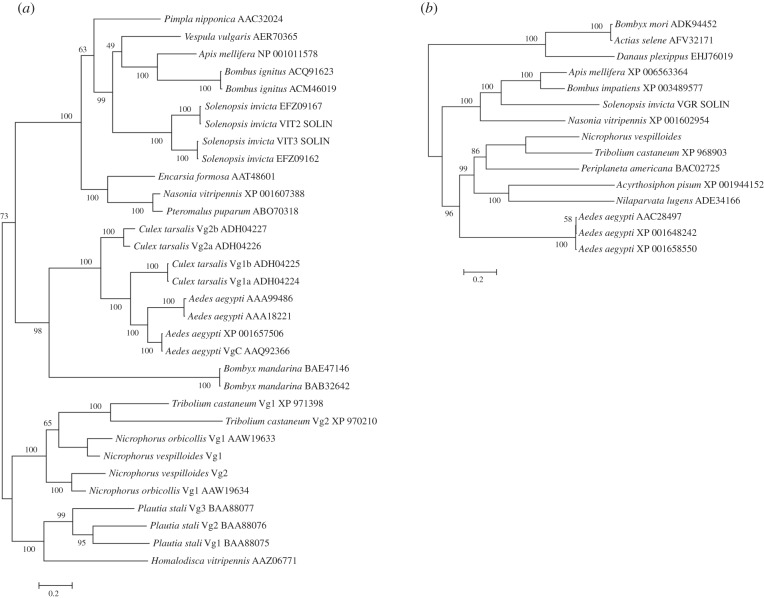
Bayesian inference phylogenetic analyses of insect Vg and VgR protein sequences. (*a*) Phylogenetic analysis using the GL/ICG domain of Vg shows that the two putative *N. vespilloides* sequences group with its respective orthologue from *N. orbicollis*. The phylogeny for VgR (*b*) has the *N. vespilloides* sequence grouping with *T. castaneum* sequence, confirming its identity. All analyses were performed using amino acid sequences and the Wag model of protein evolution.

Most insect species have multiple copies of *vg* [20,31,32]. We used genomic and transcriptome databases that we established for *N. vespilloides* to determine the copy number and obtain sequences of putative *vg* genes. The full *T. castaneum* sequences were too divergent to reliably return results from both the genome and transcriptome searches. Therefore, we had to rely on sequences published for congeneric *N. orbicollis* [21], which comprises solely an approximately 500 bp conserved domain necessary for oligomerization known as the GL/ICG motif [29,31,32]. Using *N. orbicollis* GL/ICG sequences, we were able to identify two distinct sequences from both the genomic and transcriptomic databases; however, only the most distal portions of the two conventional *vg* genes were assembled containing the majority of the GL/ICG motif for each gene. To confirm that we had obtained sequences for conventional *vg* genes, we performed phylogenetic analyses on translated sequences. These analyses showed that one of the identified *N. vespilloides* Vg groups with Vg1 from *N. orbicollis* and the other groups with Vg2 from *N. orbicollis* (figure 1*a*). This strongly suggests that there are two copies of conventional *vg* genes within *N. vespilloides* and, as expected, the duplication event predates the species split within *Nicrophorus*. For the purposes of this study, sequences that we refer to as Vg1 (KM085004) or Vg2 (KM085005) are those that group with corresponding sequences for *N. orbicollis*.

In addition to *vg* expression, we were interested in examining the expression pattern of the receptor for vitellogenin (*vgr*). Unlike *vg*, most insect species contain only a single copy of *vgr* [33]. Using published sequences from *T. castaneum* and the method described above for *vg*, we were able to obtain nearly the full sequence of *vgr* (KM085006) and phylogenetic analysis grouped the putative receptor sequence with other beetles (figure 1*b*).

### (b) Differential expression of genes during parental care

**Figure 2.**
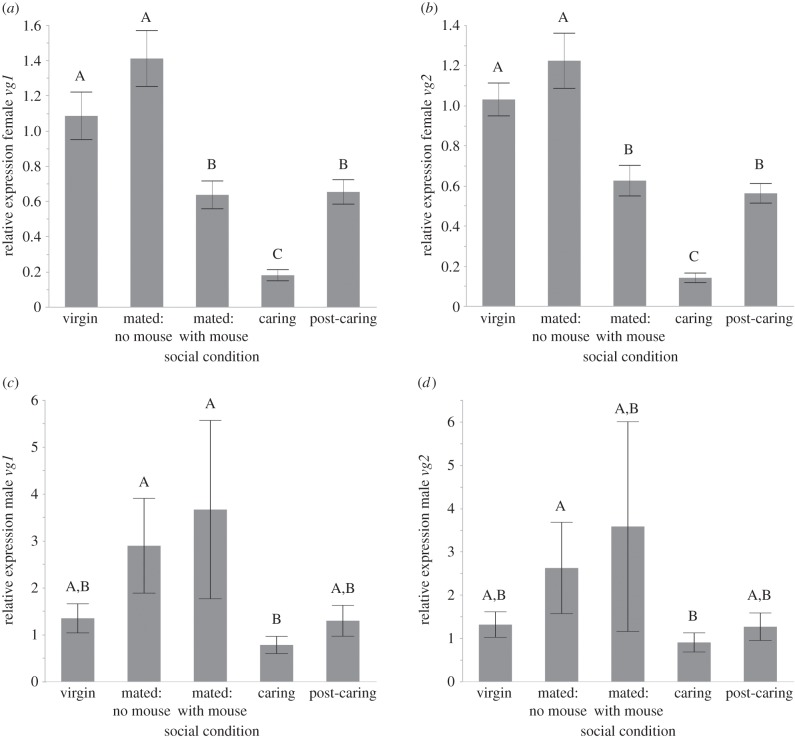
*vitellogenin* expression in head tissue of adult female and adult male *N. vespilloides* across five behavioural states. (*a*) *vg1* expression and (*b*) *vg2* expression significantly decreases during active care of larvae in females. (*c*) *vg1* expression and (*d*) *vg2* expression decreases in males during care of larvae but the reduction in expression is only statistically significant for *vg1*. Bars represent mean relative quantity and error bars represent±1 s.e.m. Bars with different letters are statistically significantly different in pairwise comparisons. Expression was measured from whole heads using 10 individuals per behavioural state.

We found that the expression pattern of *vg1* and *vg2* in head tissue changed across the five social conditions examined and that the patterns for *vg1* and *vg2* were nearly identical. There was a statistically significant association between *vg1* gene expression and social condition (figure 2*a*; *F*_4,45_ = 29.490, *p* < 0.0001) in females. There were statistically significantly lower expressions in caring females than in virgin, mated without a mouse, mated with a mouse and post-caring females (all *p* < 0.0001). There was also a statistically significantly lower expression in mated without a mouse and mated with a mouse (*p* = 0.0004) and post-caring (*p* = 0.0010). Mated with a mouse (*p* = 0.0152) and post-caring (*p* = 0.0299) were statistically significantly lower than virgin female expression. No other pairwise comparisons were statistically significant.

Expression of *vg2* was also significantly associated with social condition (figure 2*b*; *F* = 44.323, d.f. = 4,45, *p* < 0.0001). Again we found statistically significantly lower expression in caring females compared with virgin, mated without a mouse, mated with a mouse and post-caring females (all *p* < 0.0001). There was also a statistically significantly lower expression in mated without a mouse and mated with a mouse (*p* = 0.0007) and post-caring (*p* = 0.0003). Mated with a mouse (*p* = 0.0058) and post-caring (*p* = 0.0025) were statistically significantly lower than virgin female expression. No other pairwise comparisons were statistically significant.

Owing to its role as a yolk precursor protein, Vg is mainly thought of as a female-specific protein. However, in *N. vespilloides*, males perform care as well as females. Therefore, if Vg has a role in parental care and functions independently of reproduction, we predicted we would see a similar pattern of altered expression associated with caring males. We measured *vg1* and *vg2* expression in males involved in direct care under the same five social conditions described above. We found that *vg* is indeed expressed within male *N. vespilloides* and see a similar decrease in *vg* gene expression during care in males. Expression of *vg1* in males is significantly associated with social condition (figure 2*c*; *F* = 2.511, d.f. = 4, 44, *p* = 0.050). Examining pairwise comparisons, we found that *vg1* expression is significantly lower while caring for larvae compared with expression when mated without a mouse (*p* = 0.0103) or mated with a mouse (*p* = 0.0113). No other pairwise comparisons were statistically significantly different (all *p* > 0.1376). The overall pattern of expression of *vg2* was similar, decreasing during caring, but not statistically significant (figure 2*d*; *F* = 1.299, d.f. = 4,45, *p* = 0.2850).

**Figure 3.**
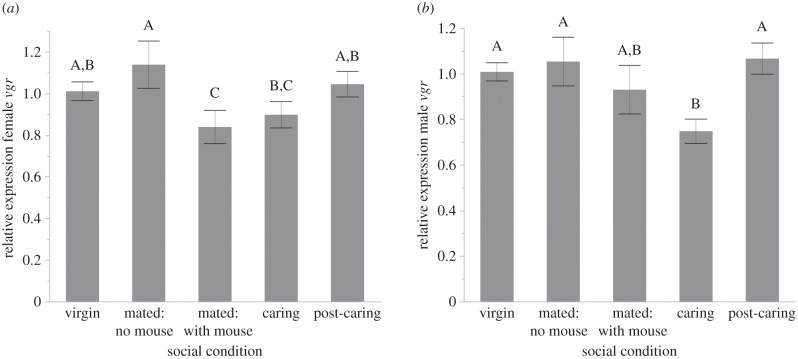
*vitellogenin receptor* is expressed in heads and isolated brain tissue of adult female and adult male *N. vespilloides*. (*a*) *vgr* expression in females shows a significant change in expression levels across the five behavioural states examined, with levels decreasing when mated on a carcass and while caring. (*b*) With one outlier removed, a similar pattern of changing expression levels of *vgr* is seen in males across different social conditions. Bars represent mean relative quantity and error bars represent±1 s.e.m. Bars with different letters are statistically significantly different in pairwise comparisons. Expression was measured from whole heads using 10 individuals per behavioural state.

We also examined the expression pattern of the receptor responsible for Vg uptake into cells. Examining *vgr* expression in head tissue of females and males across the five social conditions, we found that *vgr* was expressed at moderate levels in both females and males. Again, we see the same biological trend of decreasing *vgr* expression during caring within females (figure 3*a*; *F* = 2.765, d.f. = 4,45, *p* = 0.0390). Expression in females who were mated with a mouse was significantly lower than expression in virgin females (*p* = 0.0485), females mated without a mouse (*p* = 0.0063) and post-caring females (*p* = 0.0265). Expression in females mated without a mouse was also significantly higher than in caring females (*p* = 0.041). A similar pattern was seen for males, albeit again not as strongly and not reaching statistical significance (*F* = 2.319, d.f. = 4,44, *p* = 0.071). However, this appears to be driven by one extreme value in the ‘caring male’ state. If we exclude this value, then we do see a significant reduction in *vgr* expression during caring in males (figure 3*b*; *F* = 3.682, d.f. = 4,43, *p* = 0.0115). In caring males, expression was statistically significantly lower than in virgin (*p* = 0.0055), mated without a mouse (*p* = 0.0041) or post-caring males (*p* = 0.0017).

**Figure 4.**
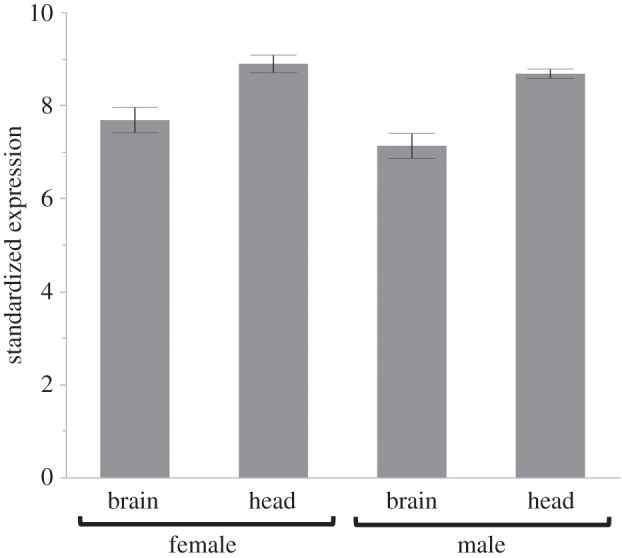
*vgr* is expressed at moderate levels in brain tissue dissected from females and males. Expression was measured using five virgin individuals per sex. Bars represent standardized −Δ*C*_T_ values (standardized so that the lowest expression = 0 and all other values expressed as deviations from this value) and error bars represent±1 s.e.m.

We measured *vgr* expression within brain tissue only and compared this to expression of whole-head extracts (including fat body and connective tissue) to confirm that Vg could be taken up within the brain and have a possible role within the brain itself and not just associated tissues in the head such as fat body. We found that *vgr* is expressed within the brains of female and male beetles (figure 4), albeit in lower levels than in the whole-head extract (*F* = 38.275, d.f. = 1,15, *p* < 0.0001). However, there was no difference in expression between males and females (*F* = 2.930, d.f. = 1,14, *p* = 0.1075), and no significant interaction between expression in the different tissue types and sex (*F* = 0.587, d.f. = 1, 14, *p* = 0.455).

## 4. Discussion

In support of our hypothesis that genes associated with reproduction will be co-opted to influence parent–offspring social interactions, we have shown that changes in *vg* and its receptor, *vgr*, were associated with changes in social behaviour in both males and females of a subsocial beetle. Vitellogenin is normally associated with its role as a yolk precursor. However, in Hymenoptera, it has also been implicated in behavioural changes and, in eusocial species, different tasks among workers. Our results suggest a role for *vg* in social interactions and parental care beyond Hymenoptera and the eusocial insects. In addition, we showed that the receptor for vitellogenin is expressed in the brains of males and females, the first study to show that *vgr* is expressed in the brain of a non-eusocial insect and in males. A decrease in *vg* expression associated with parental care in females can be explained by Vg being involved in reproduction and forgoing reproduction during care; finding a decrease in both males and females is inconsistent with Vg being involved solely in reproduction. Our results therefore support our hypothesis that the alternative role for Vg aside from reproduction can be general and help lay the framework necessary for understanding how this multifaceted protein has evolved from functioning only as a yolk precursor protein to a signal for behavioural change.

In insects, the number of conventional *vg* genes varies by species but usually ranges from one to four copies [21,31,32]. Two other beetle species, *T. castaneum* and *N. orbicollis*, possess two copies of the *vg* gene within their genome [21,34]. We therefore hypothesized that *N. vespilloides* also possesses two copies of this gene, which is what we found (figure 1*a*). Vitellogenin sequences for *N. vespilloides* are most similar to the other species of burying beetle with available sequences, and to the other sequenced beetle species *T. castaneum*. Our data suggest that the split into two conventional forms of *vg* predates the species split in *Nicrophorus*. A recent study has identified multiple homologues of Vg within insect genomes [35], but we did not identify such homologues. We also sequenced the receptor, *vgr* (figure 1*b*). Vitellogenin receptor, as the name implies, is responsible for binding circulating Vg and transporting it into oocytes through endocytosis [33]. We found a single copy of *vgr*, most closely related to other beetles and homologous to other insects, including eusocial insects.

Both male and female *N. vespilloides* beetles can provide uniparental care [17–19], providing both indirect care (carcass manipulation and maintenance), which begins even before the larvae arrive, and direct care (regurgitating food to begging offspring) once the larvae are present [30]. Consistent with both males and females as equally competent parents, we found that the pattern of *vg* expression changes in both sexes when we compare before, during and after uniparental care. Because we examined a behavioural series that reflects changes in social conditions from pre-care to post-care, we can interpret the changes in *vg* expression in light of behavioural and physiological development. In females, virgins acted as a control in our studies, as they have yet to have fully developed ovaries [36]. Once females mate, they have a social experience but still do not have fully developed ovaries. Ovaries develop and egg laying commences when females find and begin to manipulate a mouse [28,29]. This is also the point when indirect care commences. During our ‘caring’ stage, direct parenting occurs and there is no ovary development or reproduction. Post-caring reflects a cessation in parental behaviour, and a return to potential reproduction. For males, the behaviour is completely overlapping but reproductive physiology differs. Males are able to reproduce off a mouse (pass sperm during mating), and mate repeatedly before the larvae arrive [19,37], but do not mate during the active direct caring phase (i.e. when interactions with offspring occur; A.J.M. 2015, personal observation). Otherwise, the behavioural states are the same as in females. In males, juvenile hormone (JH) spikes when paired on a carcass with a mate, which stimulates the transition to parenting behaviour [36,38,39]. In females, it also stimulates ovarian development [36,38]. Thus, the five conditions we study allow us to discriminate changes associated with reproduction and care.

We found that in both females and males, there was a significant reduction in *vg* expression during caring (figure 2). We also find this in an RNA-seq study comparing caring biparental and uniparental males and females to mated controls (DJ Parker, CB Cunningham, CA Walling, CE Stamper, ML Head, EM Roy-Zokan, EC McKinney, MG Ritchie, AJ Moore, 2015, unpublished data). Again, *vg* is the most strongly differentially expressed transcript in both males and females in both biparental and uniparental conditions. This reduction in *vg* expression during care is highly suggestive of a direct role of *vg* during the behavioural transition into caring and is a significant finding in understanding the evolution of Vg's role in social behaviour. Because females do not lay eggs until they are on a resource, one might hypothesize that *vg* expression would increase during this state if Vg were solely involved in reproduction. The fact that *vg* expression decreases in female heads in our mated on a mouse samples suggests that the change in expression is not reflecting reproductive state. The presence of *vg* in males is not wholly unexpected; studies have shown that *vg* is expressed at low levels within male insects, including honeybees [29,40,41]. The change in expression during care is also seen in males, albeit with smaller changes in expression, and *vg1* shows a stronger pattern than *vg2*, which is not true of females. However, because the decreases in expression during care parallel those seen in females, our study suggests a function in affecting behaviour for male-expressed *vg*. The role of *vg* in influencing social behaviour is further supported by the presence of the receptor for Vg within head and brain tissue (figure 4), and the reduction in *vgr* expression during caring in both males and females (figure 3). This is a membrane-bound receptor that transports Vg into cells via endocytosis and thought to be expressed solely in ovaries [33]. However, more recent work suggests that other tissues may respond to Vg. Amdam *et al.* [3] showed that *vgr* is expressed in the hypopharyngeal gland in the head of honeybee workers, and Wheeler *et al.* [42] demonstrated that knocking down *vg* expression in honeybees alters overall gene expression in the brain. Both of these studies' results suggested that there should be *vgr* expressed in burying beetle heads if it has a role in behaviour, which is what we found. Overall, our results suggest a role for Vg in affecting parental care.

While our study indicates that there is an association between *vg* expression and behaviour in *N. vespilloides*, supporting the idea that reproductive genes and products are co-opted to influence parental care when reproduction does not occur, the direction of change in expression is opposite to that of honeybees. Expression of *vg* is higher when honeybee workers are caring for larvae versus when they are foraging for food. However, the change in *vg* could simply reflect changes in energy stores. Nutritional status plays a large role in behavioural maturation in honeybees, which is thought to be owing to the interactions between insulin signalling, Vg and JH [10,43]. Nursing honeybee workers have higher lipid stores than foraging workers, and this is correlated with the higher Vg levels seen in nursing workers [11,43]. As lipid stores become depleted, JH titres increase and *vg* levels decrease, resulting in a transition into foraging. The pattern of *vg* expression changes seen in *N. vespilloides* across the sampled behavioural states may reflect a similar change in lipid stores. When male and female *N. vespilloides* transition into parenting, they no longer are feeding for themselves but instead regurgitate ingested food to their offspring. The reduction in *vg* during caring may simply reflect a decrease in lipid stores within the beetle as they feed and care for their offspring. Alternatively, it may be that the decrease in *vg* expression during parental care is correlated with a decrease in aggression. Our results parallel those found in the primitively eusocial bumblebee, *Bombus terrestris*, where *vg* expression was reduced in workers even when reproductive [16]. Amsalem *et al.* [16] demonstrated that *vg* expression is decoupled from its reproductive role in *B. terrestris* and that expression levels are tied to aggressive behaviour rather than reproduction with *vg* levels—levels were highest in the most aggressive individuals but there was no difference in oocyte development between aggressive and non-aggressive individuals. In *N. vespilloides*, social tolerance is highest during parental care. The decrease in *vg* seen in *N. vespilloides* may be a reflection of an overall decrease in aggression as parents care for offspring.

Our results support the hypothesis that genes influencing reproduction are co-opted into a behavioural role [6,7,44] and suggest that co-opting genes involved in reproduction into a behavioural role extends beyond eusocial insects. The association of *vg* expression and one of the tasks of workers, parental care, in a subsocial insect also supports the heterochrony hypothesis [1,6,45], which suggests that the timing of gene expression rather than new genes will evolve to influence behaviour. While co-opting *vg* expression for other functions may be facilitated by loss of reproduction by sterile worker castes in eusocial insects, it appears that Vg may be generally associated with the evolution of social behaviour and is not specific to the evolution of eusociality.

## Data accessibility

All data from this study are publicly available in the Dryad Digital Repository: http://dx.doi.org/10.5061/dryad.hv500.

## References

[1] Linksvayer TA 2010 Subsociality and the evolution of eusociality. In Encyclopedia of animal behavior (eds Breed M Moore J), pp. 358–362. New York, NY: Academic Press.

[2] Wilson EO 1971 The insect societies. Cambridge, MA: Belknap Press.

[3] Amdam GV Norberg K Hagen A Omholt SW 2003 Social exploitation of vitellogenin. Proc. Natl Acad. Sci. USA 100, 1799–1802. (10.1073/pnas.0333979100) 12566563 PMC149913

[4] Amdam GV Norberg K Fondrk MK Page RE 2004 Reproductive ground plan may mediate colony-level selection effects on individual foraging behavior in honey bees. Proc. Natl Acad. Sci. USA 101, 11 350–11 355. (10.1073/pnas.0403073101) PMC509206 15277665

[5] Nelson CM Ihle KE Fondrk MK Page RE Amdam GV 2007 The gene *vitellogenin* has multiple coordinating effects on social organization. PLoS Biol. 5, e62 (10.1371/journal.pbio.0050062) 17341131 PMC1808115

[6] Amdam GV Page RE 2010 The developmental genetics and physiology of honeybee societies. Anim. Behav. 79, 973–980. (10.1016/j.anbehav.2010.02.007) 20514137 PMC2875690

[7] West-Eberhard MJ 1996 Wasp societies as microcosms for the study of development and evolution. In Natural history and evolution of paper-wasps (eds Turillazzi S West-Eberhard MJ), pp. 290–317. New York, NY: Oxford University Press.

[8] Nomizu T Falchuk KH Vallee BL 1993 Zinc, iron, and copper contents of *Xenopus laevis* oocytes and embryos. Mol. Reprod. Dev. 36, 419–423. (10.1002/mrd.1080360403) 8305203

[9] Montorzi M Falchuk KH Vallee BL 1994 *Xenopus laevis* vitellogenin is a zinc protein. Mol. Cell Biol. Res. Commun. 200, 1407–1413. 10.1006/bbrc.1994.1607 8185593

[10] Seehuus SC Norberg K Gimsa U Krekling T Amdam GV 2006 Reproductive protein protects functionally sterile honey bee workers from oxidative stress. Proc. Natl Acad. Sci. USA 103, 962–967. (10.1073/pnas.0502681103) 16418279 PMC1347965

[11] Corona M Velarde RA Remolina S Moran-Lauter A Wang Y Hughes KA Robinson GE 2007 Vitellogenin, juvenile hormone, insulin signaling, and queen honey bee longevity. Proc. Natl Acad. Sci. USA 104, 7128–7133. (10.1073/pnas.0701909104) 17438290 PMC1852330

[12] Guidugli KR Nascimento AM Amdam GV Barchuk AR Omholt S Simoes ZLP Hartfelder K 2005 Vitellogenin regulates hormonal dynamics in the worker caste of a eusocial insect. FEBS Lett. 579, 4961–4965. (10.1016/j.febslet.2005.07.085) 16122739

[13] Wurm Y 2010 The genome of the fire ant *Solenopsis invicta*. Proc. Natl Acad. Sci. USA 108, 5679–5684. (10.1073/pnas.1009690108) PMC3078418 21282665

[14] Corona M Libbrecht R Wurm Y Riba-Grognuz O Studer RA Keller L 2013 Vitellogenin underwent subfunctionalization to acquire caste and behavioral specific expression in the harvester ant *Pogonomyrmex barbatus*. PLoS Genet. 9, e1003730 (10.1371/journal.pgen.1003730) 23966882 PMC3744404

[15] Libbrecht R Corona M Wende F Azevedo DO Serrao JE Keller L 2013 Interplay between insulin signaling, juvenile hormone, and vitellogenin regulates maternal effects on polyphenism in ants. Proc. Natl Acad. Sci. USA 110, 11 050–11 055. (10.1073/pnas.1221781110) PMC3704040 23754378

[16] Amsalem E Malka O Grozinger C Hefetz A 2014 Exploring the role of juvenile hormone and vitellogenin in reproduction and social behavior in bumble bees. BMC Evol. Biol. 14, 45 (10.1186/1471-2148-14-45) 24618396 PMC4007805

[17] Eggert AK Müller JK 1997 Biparental care and social evolution in burying beetles: lessons from the larder. In The evolution of social behavior in insects and arachnids (eds Choe JC Crespi BJ), pp. 219–236. Cambridge, UK: Cambridge University Press.

[18] Scott MP 1998 The ecology and behavior of burying beetles. Annu. Rev. Entomol. 43, 595–618. (10.1146/annurev.ento.43.1.595) 15012399

[19] Eggert A-K 1992 Alternative male mate-finding tactics in burying beetles. Behav. Ecol. 3, 243–254. (10.1093/beheco/3.3.243)

[20] Oxley PR Ji L Fetter-Pruneda I McKenzie SK Li C Hu H Zhang G Kronauer DJC 2014 The genome of the clonal raider ant *Cerapachys biroi*. Curr. Biol. 24, 451–458. (10.1016/j.cub.2014.01.018) 24508170 PMC3961065

[21] Scott MP Panaitof SC Carleton KL 2005 Quantification of vitellogenin-mRNA during maturation and breeding of a burying beetle. J. Insect Physiol. 51, 323–331. (10.1016/j.jinsphys.2004.12.014) 15749115

[22] Lee JM Hatakeyama M Oishi K 2000 A simple and rapid method for cloning insect vitellogenin cDNAs. Insect Biochem. Mol. Biol. 30, 189–194. (10.1016/S0965-1748(99)00127-7) 10732986

[23] Larkin MA 2007 Clustal W and Clustal X version 2.0. Bioinformatics 23, 2947–2948. (10.1093/bioinformatics/btm404) 17846036

[24] Tamura K Peterson D Peterson N Stecher G Nei M Kumar S 2011 MEGA5: Molecular evolutionary genetics analysis using maximum likelihood, evolutionary distance, and maximum parsimony methods. Mol. Biol Evol 28, 2731–2739. (10.1093/molbev/msr121) 21546353 PMC3203626

[25] Ronquist F 2012 MrBayes 3.2: efficient Bayesian phylogenetic inference and model choice across a large model space. Syst. Biol. 61, 1–4. (10.1093/sysbio/sys029.) 22357727 PMC3329765

[26] Benowitz K Head ML Williams C Moore AJ Royle NJ 2013 Male age mediates reproductive investment and response to paternity assurance. Proc. R. Soc. B 280, 20131124 (10.1098/rspb.2013.1124) PMC3712429 23782889

[27] Cunningham CB Douthit MK Moore AJ 2014 Octopaminergic gene expression and flexible social behavior in the subsocial burying beetle *Nicrophorus vespilloides*. Insect Mol. Biol. 23, 391–404. (10.1111/imb.12090) 24646461 PMC4237177

[28] Trumbo ST 1997 Juvenile hormone-mediated reproduction in burying beetles: from behaviour to physiology. Arch. Insect Biochem. Physiol. 35, 479–490. (10.1002/(SICI)1520-6327(1997)35:4<479::AID-ARCH9>3.0.CO;2-M)

[29] Scott MP Trumbo ST Neese PA Bailey WD Roe RM 2001 Changes in biosynthesis and degradation of juvenile hormone during breeding by burying beetles: a reproductive or social role? J. Insect Physiol. 47, 295–302. (10.1016/S0022-1910(00)00116-5) 11119775

[30] Walling CA Stamper CE Smiseth PT Moore AJ 2008 The quantitative genetics of sex differences in parenting. Proc. Natl Acad. Sci. USA 105, 18 430–18 435. (10.1073/pnas.0803146105) PMC2587554 19008350

[31] Tufail M Takeda M 2008 Molecular characteristics of insect vitellogenins. J. Insect Physiol. 54, 1447–1458. (10.1016/j.jinsphys.2008.08.007) 18789336

[32] Hayward A Takahasi T Bendena WG Tobe SS Hui JHL 2010 Comparative genomic and phylogenetic analysis of vitellogenin and other large lipid transfer proteins in metazoans. FEBS Lett. 584, 1273–1278. (10.1016/j.febslet.2010.02.056) 20188099

[33] Tufail M Takeda M 2009 Insect vitellogenin/lipophorin receptors: molecular structures, role in oogenesis and regulatory mechanisms. J. Insect Physiol. 55, 88–104. (10.1016/j.jinsphys.2008.11.007) 19071131

[34] Sheng Z Xu J Bai H Zhu F Palli SR 2011 Juvenile hormone regulates *vitellogenin* gene expression through insulin-like peptide signaling pathway in the red flour beetle, *Tribolium castaneum*. J. Biol. Chem. 286, 41 924–41 936. (10.1074/jbc.M111.269845) PMC3234905 22002054

[35] Morandin C Havukainen H Kulmuni J Dhaygude K Trontti K Helantera H 2014 Not only for egg yolk: functional and evolutionary insights from expression, selection, and structural analyses of *Formica* ant vitellogenins. Mol. Biol. Evol. 31, 2181–2193. (10.1093/molbev/msu171) 24895411

[36] Scott MP Panaitof SC 2004 Social stimuli affect juvenile hormone during breeding in biparental burying beetles (Silphidae: *Nicrophorus*). Horm. Behav. 45, 159–167. (10.1016/j.yhbeh.2003.09.012) 15047010

[37] House CM Hunt J Moore AJ 2007 Sperm competition, alternative mating taxtics and context-dependent fertilization success in the burying beetle, *Nicrophorus vespilloides*. Proc. R. Soc. B 274, 1309–1315. (10.1098/rspb.2007.0054) PMC2176180 17360284

[38] Panaitof SC Scott MP 2006 Effect of juvenile hormone on *vitellogenin* gene expression in the fat body of burying beetles, *Nicrophorus orbicollis*. Arch. Insect Biochem. Physiol. 63, 82–91. (10.1002/arch) 16983666

[39] Panaitof SC Scott MP Borst DW 2004 Plasticity in juvenile hormone in male burying beetles during breeding: physiological consequences of the loss of a mate. J. Insect Physiol. 50, 715–724. (10.1016/j.jinsphys.2004.05.008). 15288205

[40] Trenczek TA Engels W 1986 Occurrence of vitellogenin in drone honeybees. Invertebr. Reprod. Dev. 10, 307–311. (10.1080/01688170.1986.10510254)

[41] Piulachs MD Guidugli KR Barchuk AR Cruz J Simoes ZLP Belles X 2003 The vitellogenin of the honey bee *Apis mellifera*: structural analysis of the cDNA and expression studies. Insect Biochem. Mol. Biol. 33, 459–465. (10.1016/S0965-1748(03)00021-3) 12650694

[42] Wheeler MM Ament SA Rodriguez-Zas SL Robinson GE 2013 Brain gene expression changes elicited by peripheral *vitellogenin* knockdown in the honeybee. Insect Mol. Biol. 22, 562–573. (10.1111/imb.12043) 23889463

[43] Schooley DA Baker FC 1985 Juvenile hormone biosynthesis. In Comprehensive insect physiology. Biochemistry and pharmacology (eds Kerkut GA Gilbert LI), pp. 363–389. Oxford, UK: Pergamon Press.

[44] West-Eberhard MJ 1987 Flexible strategy and social evolution. In Animal societies: theories and facts (eds Ito Y Brown JL Kikkawa J), pp. 35–51. Tokyo, Japan: Japan Scientific Societied Press, Ltd.

[45] Linksvayer T Wade MJ 2005 The evolutionary origins and elaborations of sociality in the aculeate Hymenoptera: maternal effects, sib-social effects, and heterochrony. Q. Rev. Biol. 80, 317–336. (10.1086/432266) 16250466

